# Improving de novo protein binder design with deep learning

**DOI:** 10.1038/s41467-023-38328-5

**Published:** 2023-05-06

**Authors:** Nathaniel R. Bennett, Brian Coventry, Inna Goreshnik, Buwei Huang, Aza Allen, Dionne Vafeados, Ying Po Peng, Justas Dauparas, Minkyung Baek, Lance Stewart, Frank DiMaio, Steven De Munck, Savvas N. Savvides, David Baker

**Affiliations:** 1 Department of Biochemistry, University of Washington, Seattle, WA USA; 2 Institute for Protein Design, University of Washington, Seattle, WA USA; 3 Molecular Engineering Graduate Program, University of Washington, Seattle, WA USA; 4 Howard Hughes Medical Institute, University of Washington, Seattle, WA USA; 5 Department of Bioengineering, University of Washington, Seattle, WA USA; 6 VIB-UGent Center for Inflammation Research, Ghent, Belgium; 7 Unit for Structural Biology, Department of Biochemistry and Microbiology, Ghent University, Ghent, Belgium

**Keywords:** Protein design, Protein folding, Machine learning, Proteins, Protein structure predictions

## Abstract

Recently it has become possible to de novo design high affinity protein binding proteins from target structural information alone. There is, however, considerable room for improvement as the overall design success rate is low. Here, we explore the augmentation of energy-based protein binder design using deep learning. We find that using AlphaFold2 or RoseTTAFold to assess the probability that a designed sequence adopts the designed monomer structure, and the probability that this structure binds the target as designed, increases design success rates nearly 10-fold. We find further that sequence design using ProteinMPNN rather than Rosetta considerably increases computational efficiency.

## Abstract (web summary)

Recently, a pipeline for the design of protein-binding proteins using only the structure of the target protein was reported. Here, the authors report that the incorporation of deep learning methods into the original pipeline increases experimental success rate by ten-fold.

## Introduction

Methods for designing proteins which bind with high affinity and specificity to protein targets of interest are of considerable importance in biomedicine for generating candidate therapeutics^1^, diagnostics^2^, and imaging reagents^3, 4^. Currently, the most widely used methods involve immunization of an animal with the target to elicit antibodies^5^, or screening high complexity random libraries of antibody^6^ or other scaffolds^7^ for binding activities. Although powerful, these methods require considerable experimental effort and do not provide substantial control over the properties of the resulting binding molecules. Methods for computationally designing binders could potentially provide much faster routes to affinity reagents having desired biophysical properties that target specific surface patches, and there has been considerable progress in computational design of protein binding proteins based on extension of binding motifs observed in protein structures^8–12^. Recently, a general Rosetta-based approach to designing binding proteins using only the structure of the target was developed and used to design binding proteins to 13 different target sites^13^. Given a specified region on a target of interest, the method designs sequences predicted to fold up into protein structures that have shape and chemical complementarity to the region. While providing a general computational route to designing binders to arbitrary protein targets, the method requires screening of large numbers of computationally designed binders to identify hits as only a small fraction typically have sufficiently high affinity for experimental detection.

In parallel with advances in physical model based protein binder design, deep learning methods have achieved unprecedented accuracy in protein structure prediction. In contrast to Rosetta and other physically based molecular mechanics methods, which employ energy functions with one or two thousand parameters obtained from structural and thermodynamic data on proteins and small molecules^14^, the deep learning structure prediction methods AlphaFold2^15^ (AF2) and RoseTTAFold^16^ (RF) have hundreds of millions of parameters obtained by training on very large datasets of protein sequences and structures, and make no assumptions about pairwise decomposability or functional form. In place of the energy-guided stochastic conformational sampling approaches utilized by physically based approaches – molecular dynamics in many protein dynamics studies or Monte Carlo plus minimization in the case of Rosetta– the deep learning methods learn iterative transformations of representation of the sequence and possible structure that very rapidly converge on often quite accurate models (the successive transformations are analogous to the structure updates in traditional simulation, but are more concerted, more directed to the likely correct structure, and there is a more accurate stopping criterion^17^). For accurate prediction of the structures of naturally occurring proteins, both AF2 and RF generally require multiple sequence alignments (which contain rich co-evolutionary information on residues likely to be in contact, etc), but for de novo designed sequences, which are generally more stable and more regular than naturally occurring proteins, accurate predictions can be obtained from single sequences^18, 19^. There has also been progress in accuracy prediction for protein structure models; for example DeepAccuracyNet (DAN), which uses a representation consisting of 3D convolutions of local atomic environments^20^, achieved state-of-the-art performance in accuracy prediction in CASP14.

**Fig. 1:**
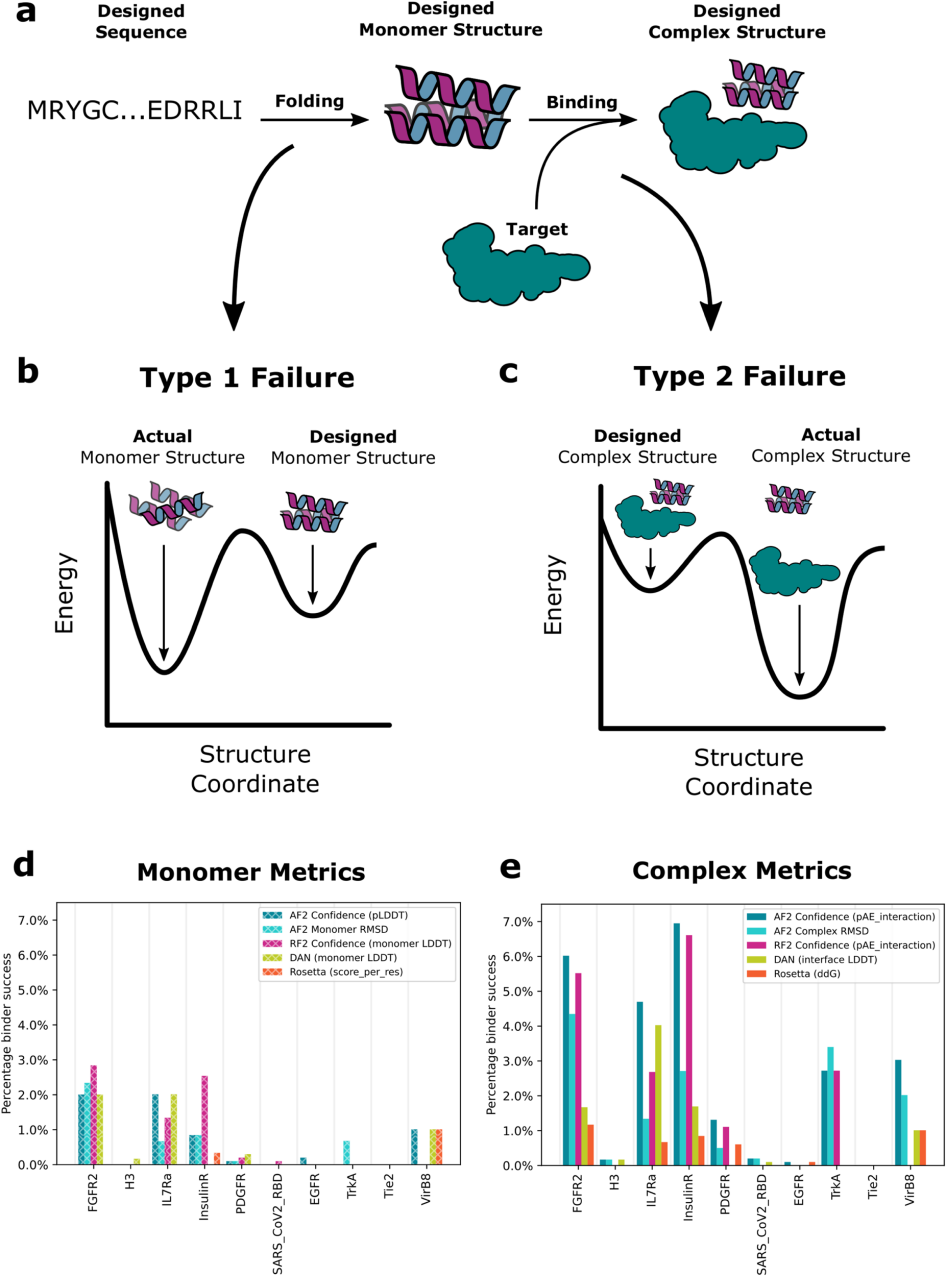
Monomer and protein complex structure prediction metrics distinguish previously designed binders from non-binders. **a** For binder design to be successful, the designed sequence must fold to the designed binder monomer structure (left), and this structure must form the designed interface with the target protein (right). **b**, **c** Design failure modes. **b** Type-1 Failures. The designed sequence does not fold to the designed monomer structure. **c** Type-2 Failures. The designed sequence folds to the designed monomer structure but does not form the designed interface. **d**, **e** The retrospective experimental success rate (YSD SC_50_ < 4 μM) for the top 1% of designs selected according to different monomer (**d**) or protein complex (**e**) based m**e**trics over 10 targets from Cao et al. Source data are provided as a Source Data file.

We reasoned that these newly-developed DL methods could increase the success rate of Rosetta-based protein binder design. As noted above, while providing a general computational route to designing binders to arbitrary protein targets, the overall success rate is quite low. The approach has two primary failure modes (Fig. 1a): first, the designed sequence may not fold to the intended monomer structure, and second, the designed monomer structure may not actually bind the target (Fig. 1b). The physically based Rosetta approach frames both the folding and binding problems in energetic terms; for the approach to succeed, the designed sequence must have as its lowest energy state in isolation the designed monomer structure, and the complex between this designed monomer structure and the target must have sufficiently low energy to drive formation of the design-target protein complex. The primary challenges in accurate design of both the monomer structure and the protein-protein interface are inaccuracies of the energy function which for computational tractability is generally represented as a sum of pairwise decomposable terms (in Rosetta: Lennard Jones, hydrogen bonding, electrostatic, solvation, and bonded geometry), and the very large size of the space which must be sampled; if the energy function is inaccurate, or conformational sampling is incomplete, the designed sequence may not fold to the intended monomer structure and/or the monomer may not bind to the target as intended.

In this work, we develop a deep learning-augmented de novo protein binder design protocol. We show retrospectively and prospectively that this improved protocol has nearly 10-fold higher success rate than the original energy-based method.

## Results

### Retrospective analysis of type I failures

We began by investigating the ability of deep learning methods to discriminate binders from non-binders (a task we call filtering) in the set of ~1 million experimentally characterized designs for 10 different targets described in Cao et al. 15,000–100,000 designs were experimentally tested for each target, and the number of actual binders ranged from 1 to 584.

We first focused on identifying Type I failures (Fig. 1) in which the designed sequence does not fold to the intended monomer structure. As a baseline, we used the Rosetta energy of the monomer, normalized by chain length (since energy is an extensive quantity). Not surprisingly as this metric was already used as a stringent filter in generating the input scaffold set for the Rosetta interface design calculations^21^, it provided little discriminatory power (Fig. 1d). In contrast, the deep learning-based accuracy prediction method DAN was able to partially discriminate binders from non-binders (Fig. 1d).

While DAN is very fast, taking ~0.5 GPU seconds per monomer structure, AF2 structure predictions are relatively slow (~5 GPU seconds). As an initial test of the utility of AF2 for monomer structure modeling, we evaluated the ability of AF2 to predict the structures of the binder monomers for the five minibinder structures from Cao et al. for which structures have been solved experimentally (for designs in complex with TrkA, FGFR2, IL-7Rɑ, and the SARS-CoV-2 Spike protein). Given only the single sequence for the designed binder, AF2 predicted the monomer structure with binder Cɑ accuracy between 0.2 Å−0.8 Å for all binders except for LCB1 which was predicted with 1.5 Å accuracy (Supplementary Fig. 1). An updated version of RoseTTAFold (RF2^22, 23^) was also found to predict all monomer structures with binder Cɑ accuracy between 0.2 Å−0.8 Å, except for TrkA which was predicted with 1.8 Å accuracy (Supplementary Fig. 1).

Encouraged by this accuracy, we set out to filter the entire set of Cao et al. designs based on the similarity of the AF2 or RF2 predicted monomer structure to the designed structure (disagreement is an indication of a possible Type I failure). For each designed sequence for each target, using AF2 or RF2 with a single sequence as input, we predicted the structure of the binder monomer. We found that the closer the prediction of the binder structure was to the Rosetta-designed structure in Cɑ RMSD, the more likely a binder was to be successful (Supplementary Fig. 4). We also found that the prediction confidence metric pLDDT was predictive of success (Fig. 1d); the two metrics are quite correlated (Supplementary Fig. 5; the pLDDT of AF2 and RF2 were equally discriminative). These results suggest that Type I failures contribute to the low success rate of binder design, and that such failures can, in part, be identified by discrepancies between design models and AF2 or RF2 structure predictions.

### Retrospective analysis of type II failures

To estimate the likelihood of the designed binder structure forming an interface with the intended target, Cao et al. primarily used the difference in energy of the bound complex and the unbound monomers allowing sidechain repacking as computed by Rosetta (Rosetta ddG), and despite the extensive use of this metric during the original calculations, Rosetta ddG remains an effective filter (Fig. 1e). We investigated the efficacy of DAN in supplementing Rosetta in assessing the accuracy of the designed complex structure. We found DAN’s complex accuracy metric to be approximately as predictive of binder success as Rosetta ddG (Fig. 1e).

We next investigated whether AF2 and RF2 complex prediction could be used to discriminate designs that form the intended complex structure from those that do not. We again began by evaluating the ability of AF2 and RF2 to reproduce the five experimentally determined minibinder structures from Cao et al. Given an MSA for the target protein and the single sequence of the designed binder, AF2 predicted the complex structure with binder Cɑ accuracy between 1.0Å−2.0 Å for three of five, and RF2 for four of five. The two structures that were not correctly predicted by AF2 were LCB1 and LCB3 which both target the SARS-CoV-2 Spike protein; AF2 was not able to correctly model a long loop in the Spike protein which caused the binders to be predicted as unbound. RF2 also predicted LCB1 as unbound (Supplementary Fig. 1). To enable AF2 to be used for binding prediction in cases where the target is incorrectly modeled, we investigated providing the target structure to the model as a template. We found this allowed AF2 to predict the correct COVID spike structures but caused all of the interfaces except FGFR2 to be predicted incorrectly (Supplementary Fig. 1). We next investigated initializing the AF2 pair representation with an encoding of the Rosetta binder structure; we call this protocol “AF2 initial guess” (see AF2 Initial Guess in Methods). Using AF2 with target template and an initial guess, AF2 is able to recapitulate the experimentally determined structures for all 5 minibinder interfaces with binder Cɑ accuracy between 1.0Å−2.0 Å RMSD (Supplementary Fig. 1). Notably, for all structures except LCB1 and LCB3, the AF2-predicted structures are closer to the experimentally determined structure than the original design models, even after extensive relaxation using Rosetta.

We used the AF2 initial guess approach and RF2 without a starting model to generate complex models for each designed sequence for each target, and compared the predicted structure of the complex to the designed complex structure. The Cɑ RMSD of the predicted complex to the Rosetta-designed complex model was predictive of design success in both cases (Fig. 1e). We obtained the best discrimination of binders from non-binders using the pAE prediction confidence metrics produced by the two methods (Fig. 1e). For the IL7Ra, TrkA, FGFR2, InsulinR, and PDGFR datasets from Cao et al, the average pAE of interchain residue pairs (pAE_interaction) was extremely effective in identifying the experimentally confirmed binders (Fig. 1e); confident predictions had very high success frequencies (see the Receiver Operator Characteristic (ROC) curves in Supplementary Fig. 6) with sharp increases in success rates for designs with pAE_interaction <10. AF2 had slightly better performance than RF2 (Fig. 1e), and we used this in the new design campaigns described in the following section. The excellent performance of both AF2 and RF2 on the binder discrimination task strongly suggest that Type II errors are primarily responsible for the low success rates of Cao et al.

### Prospective analysis

**Fig. 2:**
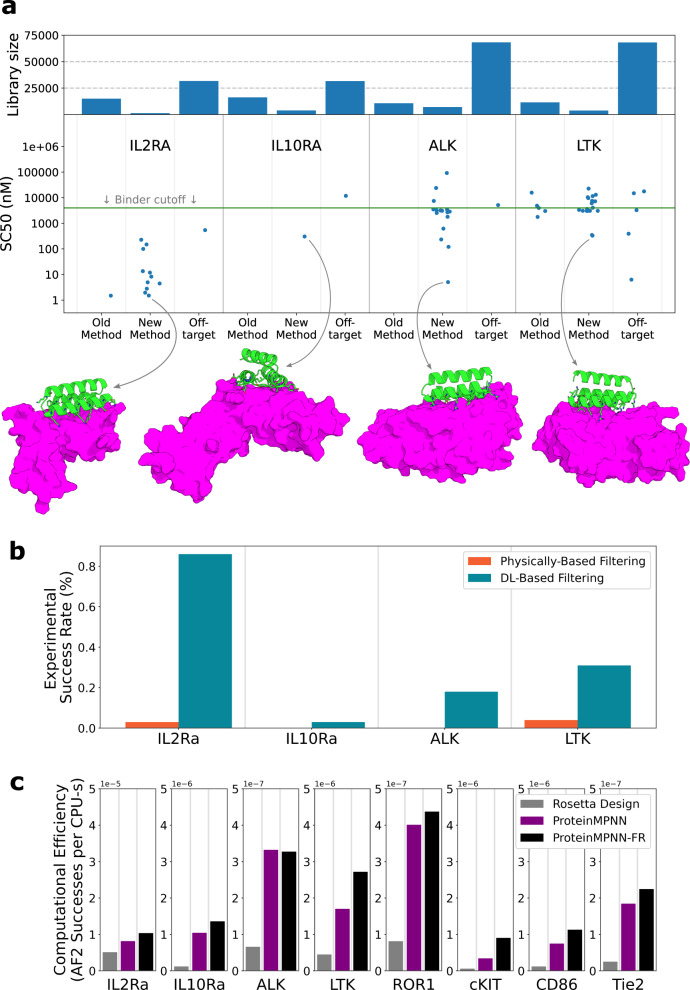
Incorporation of structure prediction metrics increases design success rate on new targets. **a** Results of Prospective Campaigns. For each target the SC_50_ from YSD is shown for all designs which showed binding by YSD (like Kd’s, lower values are better). The number of designs included in each library for each target is indicated by the bars in the top panel. The AF2-predicted structure of the top scoring on-target design is shown as a cartoon. No binders were identified to Site 2 of IL2 receptor-ɑ so this campaign is not included here or in panel C. **b** The experimental success rate for libraries filtered by DL-based filtering versus Physically based filtering for the four prospective targets. **c** The computational efficiency (the number of designs with pAE_interaction <10 per CPU-s) for the ProteinMPNN sequence design plus Rosetta relax protocol outperforms that of the original Rosetta sequence design protocol. Source data are provided as a Source Data file.

The retrospective analysis in Fig. 1 suggests incorporation of AF2 or RF2 into the design pipeline as a final evaluation filter could considerably increase the design success rate. To directly test this hypothesis, we carried out binder design campaigns on four targets of considerable biological importance: ALK^24^, LTK^24^, IL10 receptor-ɑ (IL-10Rɑ)^25^, and IL2 receptor-ɑ (IL-2Rɑ)^26–29^. As is clear from the retrospective analysis of the Cao et al. data (Fig. 1d, e), binder success rate and the predictivity of metrics varies between targets: generating designs for new targets (where there is no a priori knowledge of which filters would be predictive) is the most unbiased approach for comparing different design protocols. For IL-2Rɑ, two separate sites were targeted with independent campaigns. Using the Rosetta-based design protocol of Cao et al., we generated computational libraries of ~2 million designs for each target and filtered these down to ~20,000 designs to be experimentally tested for each target: ~15,000 designs using the physically based filters of Cao et al. and ~5000 designs with AF2 pAE_interaction <10 (these designs were also filtered by additional metrics as described in the Supplement). Synthetic genes were obtained for the ~80,000 designs, transformed into yeast, and the resulting library sorted for display of the proteins on yeast cells, followed by sorts at 1 μM target with avidity, and sorts at decreasing concentrations of target. The frequency of each design at each sort was determined by deep sequencing, and SC_50_ values (the concentration where half of the expressing yeast-cells are collected) estimated as described in Cao et al. Designs with SC_50_ values better than 4 μM were considered successes; the number of successes for the four targets ranged from 1 to 17. For each target, several designs found to bind by YSD were expressed in E. coli and binding was confirmed by single-concentration Biolayer Inferometry (BLI). All designs which showed binding by YSD also showed binding by BLI (Supplementary Fig. 9; for IL-10Rɑ where only one binder was identified, only this single design was screened by BLI). For all four targets, there was a considerably higher success rate (number of successes / number of designs tested) in the AF2-filtered design set than in the Rosetta set (Fig. 2). Physically based filtering yielded successful binders for two targets: LTK and Site 1 of IL-2Rɑ; for these the AF2-filtered libraries had 8- and 30-fold higher success rates, respectively. AF2-filtered libraries also yielded successful binders to both ALK and IL-10Rɑ; physically based filtering yielded no successful binders to either of these targets (Neither filtering method was able to generate successful binders to Site 2 on IL-2Rɑ). Thus, AF2 filtering performs as expected in prospective tests, increasing success rates (for targets where physically based filtering is successful) and expanding the set of targets for which successful minibinders can be generated.

#### Increasing binder design pipeline compute efficiency with ProteinMPNN

While an effective predictor of binder success, the AF2 filter is computationally expensive (~30 GPU-seconds per design) and only ~2.3% of designs pass, so large numbers of prediction calculations must be run. To enable the testing of large (~5,000) pools of designs, it is desirable to decrease the computational demand of the design pipeline, in particular to maximize the number of designs passing the AF2 filter a method can generate per unit compute time (the time to generate all designs and run AF2; we use a conversion factor of 100 CPU-s to 1 GPU-s because of the relative scarcity of GPU resources).

1 $${Efficiency}={Success}\,{Rate}*\,{Throughput}=	\frac{{Number}\,{of}\,{Designs}\,{with}\,{pAE}{{{{{\rm{\_}}}}}}{interaction}\, < \,10}{{Total}\,{Number}\,{of}\,{Generated}\,{Designs}} \\ 	\ast \frac{1}{{Compute}\,{Time}\,{to}\,{Generate}\,{One}\,{Design}}$$

Using this metric, we find that Rosetta-design has an efficiency of about 7.6×10^−7^ successful designs per CPU-s equivalent.

We investigated whether the recently developed deep learning graphical model based sequence design method ProteinMPNN^30^ could be used to increase the efficiency of the design pipeline. ProteinMPNN is very fast, generating a sequence for a minibinder backbone in ~2 CPU-s compared to ~350 CPU-s for Rosetta-design. We first compared the experimental success rate of ProteinMPNN designs to Rosetta designs by generating sequences for backbones generated by AF2 for Rosetta designs to the four new targets that had low complex Cɑ RMSD to the AF2 prediction (~10^4^ designs in total). Genes encoding designs with AF2 pAE_interaction <10 (~10^3^ per method) were synthesized, and the binding evaluated by FACS followed by deep sequencing as described above. For each target, several designs from ProteinMPNN were expressed in E. coli and their binding was verified with BLI, we again found that all designs which bound by YSD showed binding by BLI (Supplementary Fig. 9). We found that the design success rate of ProteinMPNN and Rosetta-design were similar (Supplementary Fig. 7), thus the considerable increase in speed comes with no decrease in performance.

Encouraged by the speed and performance of ProteinMPNN design, we next evaluated its efficiency in generating sequences passing the AF2 cutoffs. ProteinMPNN design alone had an efficiency of 1.6 × 10^−6^ successful designs per CPU-s equivalent.  The average of the fold efficiency improvement over all targets is ~5-fold greater for ProteinMPNN compared to Rosetta-design (Fig. 2c). Since unlike Rosetta, ProteinMPNN keeps the protein backbone fixed, it is sensitive to the input backbone structure quality. Inspired by the very efficient alternation between sequence optimization and structure refinement in Rosetta flexible backbone design^31^, we evaluated similar cycling between ProteinMPNN and Rosetta structure refinement (FastRelax), hoping to converge on a high-quality backbone that would then allow ProteinMPNN to generate a high-quality sequence. This hybrid ProteinMPNN/Rosetta sequence design protocol (henceforth referred to as ProteinMPNN-FR) generated AF2 pAE_interaction <10 structures at a rate of ~6.6% with a throughput of 1 design per 120 CPU-s for an efficiency of 2.2×10^−6^. The average per-target efficiency improvement of ProteinMPNN-FR over Rosetta-design is ~8-fold (Fig. 2c).

## Discussion

These experiments show that by complementing physically based methods with deep learning-based approaches trained on large numbers of protein structures, significant improvements to the one-sided protein-interface design challenge can be achieved. Our retrospective and prospective studies suggest an increase in design success rate of ten fold. In contrast to Rosetta energy calculations and DAN structure accuracy measures, which operate on single protein structures (or with Rosetta relax calculations, structures very close to the query), structure prediction calculations implicitly assess the fit of the sequence with the desired target structure compared to all others. As observed previously^32^, such consideration of the overall folding landscape enables considerably more accurate assessment of the likelihood a design will fold and bind as intended compared to evaluation of only the depth of the designed energy well. Although the protocol reported here is an order-of-magnitude improvement over the previous state-of-the-art, it is clear that much about interface energetics remains poorly understood; success rates among the targets remain low (<1%) and no binders were identified to Site 2 of IL2 receptor-ɑ. There is also considerable room for improvement in designing high affinity; as with the original pipeline the initially generated binders are in the high nM affinity range. Given the rate of progress in the field, we anticipate further increases in design success rates and affinities in the near future, which will make computational protein design methods even more powerful compared to empirical selection methods for generating affinity reagents and therapeutic candidates. While continued progress is nearly certain, an open question is whether this will come from integration of deep learning and physically based methods, or from deep learning alone–there are exciting times ahead!

## Methods

### AF2 initial guess

The protein structure provided to the model as an initial guess is first converted to AlphaFold atom positions. These positions are then provided, along with the standard model inputs into the AlphaFold Model Runner. In the AlphaFold class of the AlphaFold code, on the first recycle, the prev_pos variable is initialized to the input AlphaFold atom positions as opposed to the standard initialization of all zeros. A script to run AF2 with an initial guess and the modified source code is provided here: https://github.com/nrbennet/dl_binder_design^33^. The AlphaFold model used in the script and in this work is configured to run with a reduced number of extra MSA sequences which speeds the inference of the network dramatically, as described in previous work^34^.

### ProteinMPNN FastRelax

This protocol takes as input a protein complex structure. ProteinMPNN is then provided the complex structure with the binder sequence masked and asked to assign the binder a sequence. The new sequence is then threaded back onto the binder structure in the complex and the complex structure is relaxed using Rosetta FastRelax. The relaxed complex structure can then be used as the input to ProteinMPNN to continue the cycle. A python script to perform this design technique is provided here: https://github.com/nrbennet/dl_binder_design^33^.

### Design and filtering procedure for prospective study

The prospective study was performed at a time of rapid protocol discovery with a tight deadline for placing the gene-order. As such, not every experiment that could have been performed was performed. However, the comparison of Rosetta filtering to AF2 filtering was the main goal and the data required for this comparison was plentiful.

The standard procedure from Cao et. al. was followed for the 4 targets starting with the following pdbs: IL2RA “1Z92”, “2B5I”, “3NFP”, “2ERJ”), IL10RA (“1LQS”), ALK (Privately communicated structure. Now “7NWZ”), LTK (Privately communicated structure. Now “7NX0”). The “recommended_scaffolds.list” from Cao et. al. were used and on the order of 10 M RifDock^35^ outputs were generated for each target with about 500 K FastDesigned. ~6 K motifs were extracted, grafted up to 10 M docks, and 500 K FastDesigned again. The resulting 1 M designs for each target were predicted by AF2.

From this set of 1 M designs, 3 overlapping subsets were selected. The first subset was the Rosetta-control group where the AF2 predictions were ignored and the top ~18 K per target were selected by the pareto-front method from Cao et al. looking at target_delta_sap, ddG, contact_patch, and contact_molec_sq5_apap_target. The second subset was the AF2-filtered group where all designs passing pae_interaction <10 and af2_complex_rmsd <5 Å were included. This set was typically around 8 K per target. The third subset was all predictions with af2_complex_rmsd <5 Å. These designs were designated to be redesigned and were typically about 12 K in scale.

These AF2-predicted interfaces were then designed either with Rosetta or ProteinMPNN. Here, ProteinMPNN was used to generate a protein sequence from the input coordinates and no further optimization was performed. The Rosetta-redesigned and ProteinMPNN-redesigned pools were predicted again by AF2 and were filtered either with the Rosetta filters mentioned above or the AF2 filters mentioned above resulting in pools of sizes 9 K (Rosetta-Rosetta), 2 K (Rosetta-AF2), and 2 K (ProteinMPNN-AF2). The Rosetta filters weren’t used to filter ProteinMPNN designs because Rosetta models of ProteinMPNN outputs didn’t exist.

### DNA library preparation

DNA libraries were prepared in the manner described in Cao et al., we review this protocol here:

The sequences of protein designs were padded to 65 amino acids through addition of a (S)n linker at the C-terminus. The protein sequences were reverse translated and codon optimized for *Saccharomyces cerevisiae* using DNAworks2.0^36^. After reverse translation, DNA adapter sequences are added to the N (GGTGGATCAGGAGGTTCG) and C (GGAAGCGGTGGAAGTGG) terminus. Designs were purchased as oligonucleotide libraries from Agilent Technologies.

Oligonucleotide libraries were amplified using Kapa HiFi polymerase (Kapa Biosystems) with a qPCR machine (Bio-Rad, CFX96). The PCR product was run on a DNA agarose gel, the band with the correct size was cut out of the gel and cleaned (Qiagen QIAquick Clean up kit). The extracted DNA products were then re-amplified and purified following the above protocol. The resulting DNA inserts and linearized pETcon3 vector were transformed into EBY100 yeast following an established protocol^37^.

To prepare libraries for deep sequencing, yeast plasmids were isolated from 5 × 10^7^ to 1 × 10^8^ yeast cells by Zymoprep (Zymo Research). Two qPCR amplifications were then performed following the protocol in the above paragraph. Illumina adapters and 6-bp pool-specific barcodes were added in the second amplification. The final DNA product was purified by gel extraction. The libraries were sequenced using Illumina NextSeq sequencing.

### Yeast surface display

Yeast surface display experiments were performed in the manner described in Cao et al., we review this protocol here:

EBY100 yeast were grown in C-Trp-Ura media supplemented with 2% (w/v) glucose. Yeast cells were centrifuged and resuspended in SGCAA media supplemented with 0.2% (w/v) glucose. Cells were resuspended to a concentration of 1×10^7^ cells per ml and induced at 30°C for 16-24 hours. Cells were washed with PBSF (PBS with 1% (w/v) BSA) and then labeled with biotinylated target. To allow for the identification of low affinity binders, an initial sort with target avidity was performed for all libraries. In the avidity sort, the cells are incubated with biotinylated target, anti-c-Myc fluorescein isothiocyanate (FITC, Miltenyi Biotech) and steptavidin-phycoerythrin (SAPE, ThermoFisher). To allow all SAPE molecules to display four biotinylated target molecules, the biotinylated target is provided at a 4x excess over the concentration of SAPE. When sorting without avidity, the cells are incubated first with biotinylated target alone, then washed in PBSF and subsequently incubated with SAPE and FITC. Each library was sorted against a titration of target concentrations. Sorts were performed using a Sony SH800S cell sorter with software version 2.1.5.

### Protein expression

Proteins were expressed and purified in the manner described in Cao et al., we review this protocol here:

Genes encoding the designed protein sequences were purchased from Integrated DNA Technologies (IDT). All genes included an N-terminal 8-His tag followed by a TEV cleavage site. The genes were cloned into modified pET-29b(+) *E. coli* plasmid expression vectors. Plasmids were transformed into chemically competent *E. coli* BL21(DE3) cells (NEB). Cells were either grown overnight in Studier autoinduction media supplemented with antibiotics or induced using the IPTG expression system and then grown overnight. Cells were then lysed by sonication and the protein samples were purified by immobilized metal affinity chromatography (Qiagen) followed by size-exclusion fast protein liquid chromatography (Superdex 75 10/300 GL, GE Healthcare).

### Target protein preparation

#### Expression and purification of biotinylated ALK and LTK ectodomains

DNA encoding for the cytokine binding domains of ALK (ALK_TG-EGFL_, residues 648-1030) and LTK (LTK_TG-EGFL_, residues 63-420) were cloned in the pHLsec vector in frame with a N-terminal chicken RTPμ-like signal peptide sequence and a C-terminal Avi-tag followed by a caspase-3-cleavable Fc-His_x6_ tag^38^.

Proteins were produced in HEK293S suspension cells maintained in growth medium consisting of 50% Freestyle (Thermofisher) and 50% Ex-Cell (Sigma-Aldrich). Transient transfection was performed using linear 25 kDA polyethyleneimine (Polysciences) as transfection reagent. To allow specific in vivo biotinylation of the Avi-tag, both constructs were co-transfected with the pDisplay-BirA-ER plasmid in a 4:1 pHLsec:pDisplay stoichiometric ratio^39^. The growth medium was supplemented with D-biotin to a final concentration of 100 μM to ensure complete biotinylation of the recognition sequence. After 4 days of expression, conditioned medium was clarified by centrifugation and filtered through a 0.22 μm filter prior to chromatographic steps.

Proteins were captured via their Fc tag on a protein A column (HiTrap Protein A HP, Cytiva) and eluted in HBS (20 mM HEPES, pH 7.4, 150 mM NaCl) after an on-column digestion with caspase-3 for 1 h at 37 °C and an additional 2-h incubation at room temperature. As a final polishing step, recombinant proteins were concentrated and injected onto a Superdex 200 increase 10/300 GL (Cytiva) size-exclusion chromatography column pre-equilibrated with HBS. Purified biotinylated proteins were flash frozen in liquid nitrogen and stored at −80 °C until further use.

Biotinylated IL-10Rɑ was purchased from R&D Systems (AVI9044). Biotinylated IL-2Rɑ was purchased from Acro Biosystems (ILA-H82E6).

### Biolayer interferometry binding experiments

Biolayer interferometry (BLI) measurements were performed on an Octet Red96 (ForteBio) or Octet R8 (Sartorius) instrument with Octet BLI Discovery 12.2.1.18 software, with streptavidin coated tips (Sartorius Item no. 18-5019). The binding buffer consisted of 1X HBS-EP + buffer (Cytiva BR100669) supplemented with 1.0% w/v bovine serum albumin. 30-50 nM (depending on target availability) of target protein was loaded onto the tips. After target loading, a baseline measurement was performed in binding buffer alone for 120 s. The tips were then dipped in a solution of 500 nM (1000 nM for the IL-10Rɑ design) protein analyte in binding buffer for 600 s (association phase). The tips were then dipped back into binding buffer alone for 1000 s (dissociation phase).

### Statistics and reproducibility

No statistical method was used to predetermine sample size. No data were excluded from the analyses. The experiments were not randomized. The Investigators were not blinded to allocation during experiments and outcome assessment.

### Reporting summary

Further information on research design is available in the Nature Portfolio Reporting Summary linked to this article.

## Supplementary information

Supplementary Information

Peer Review File

Reporting Summary

## Data Availability

The raw data from the prospective study, the raw scores of the retrospective analysis, the input structures and benchmarking scores for the efficiency study, and the raw data from the biolayer interferometry measurements are available at the following repository hosted by the Institute for Protein Design: The main supplement (136 MB) Contains these files: design_models_final_combo_optimized/ design_models_sequence/ design_models_ssm_natives/ design_stats/ dna_production_scripts/ figure_data/ ngs_analysis_scripts/ files.ipd.uw.edu/pub/improving_dl_binders_2023/supplemental_files/scripts_and_main_pdbs.tar.gz Experimental data and data derived from that data (155 MB) Contains these files: ngs_data/ ngs_data_analysis/ files.ipd.uw.edu/pub/improving_dl_binders_2023/supplemental_files/experimental_data_and_analysis.tar.gz All ordered proteins in.pdb.gz format: (~100 K files; 15 GB) Contains these files: design_models_pdbs/ files.ipd.uw.edu/pub/improving_dl_binders_2023/supplemental_files/design_models_pdb.tar.gz All ordered proteins in Rosetta binary silent format (6.1 GB) Contains these files: design_models_silent/ files.ipd.uw.edu/pub/improving_dl_binders_2023/supplemental_files/design_models_silent.tar.gz The docks we used for the efficiency benchmark (6.1 GB) Contains these files: efficiency_benchmark_docks/ files.ipd.uw.edu/pub/improving_dl_binders_2023/supplemental_files/efficiency_benchmark_docks.tar.gz. Source data are provided with this paper.

## Source data

Source Data File

## Code availability

The Rosetta macromolecular modeling suite (https://www.rosettacommons.org) is freely available to academic and non-commercial users. Commercial licenses for the suite are available via the University of Washington Technology Transfer Office. Scripts for running ProteinMPNN-FastRelax and AF2 with templating and initial guess are available at https://github.com/nrbennet/dl_binder_design^33^.
