# Supplementary material for: Improving de novo protein binder design with deep learning
Source: Nat Commun. 2023 May 6;14:2625. doi: 10.1038/s41467-023-38328-5 (PMC10163288; doi:10.1038/s41467-023-38328-5)
Supplement: Supplementary file 1 — Supplementary Information [file 41467_2023_38328_MOESM1_ESM.pdf]

Supplementary Information for

Improving *de novo* Protein Binder Design with Deep Learning

Nathaniel R. Bennett[1,2,3‡], Brian Coventry[1,2,4‡], Inna Goreshnik[1,2], Buwei Huang[1,2,5], Aza Allen[1,2], Dionne Vafeados[1,2], Ying Po Peng[1,2], Justas Dauparas[1,2], Minkyung Baek[1,2], Lance Stewart[1,2], Frank DiMaio[1,2], Steven De Munck[6,7], Savvas N. Savvides[6,7], David Baker[1,2,4*]

* Corresponding author. Email: dabaker@uw.edu

Supplementary Figures

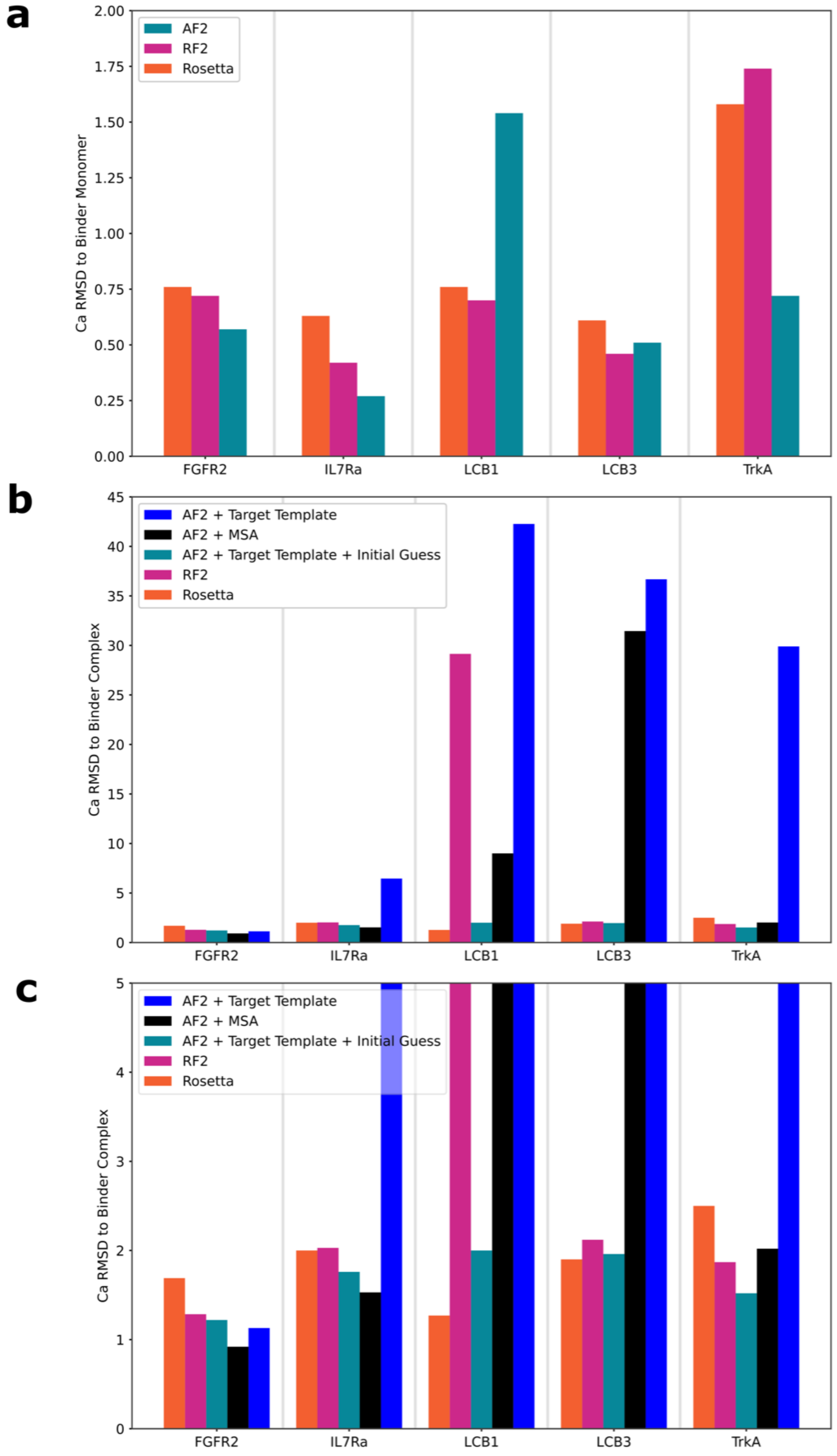
a
AF2
RF2
Rosetta
Ca RMSD to Binder Monomer
2.00
1.75
1.50
1.25
1.00
0.75
0.50
0.25
0.00
FGFR2
IL7Ra
LCB1
LCB3
TrkA
b
AF2 + Target Template
AF2 + MSA
AF2 + Target Template + Initial Guess
RF2
Rosetta
Ca RMSD to Binder Complex
45
40
35
30
25
20
15
10
5
0
FGFR2
IL7Ra
LCB1
LCB3
TrkA
c
AF2 + Target Template
AF2 + MSA
AF2 + Target Template + Initial Guess
RF2
Rosetta
Ca RMSD to Binder Complex
5
4
3
2
1
0
FGFR2
IL7Ra
LCB1
LCB3
TrkA

Supplementary Figure 1. **AF2 Prediction of Experimentally Determined Minibinder Structures.** **a** The accuracy in Cα RMSD of AF2 and RF2 predictions of binder monomer structure for the five minibinder complex structures reported in Cao et al. **b** The accuracy in Cα RMSD of AF2 and RF2 predictions of binder complex structure for the five minibinder complex structures reported in Cao et al. **c** A close-up view of B. Source data are provided as a Source Data file.

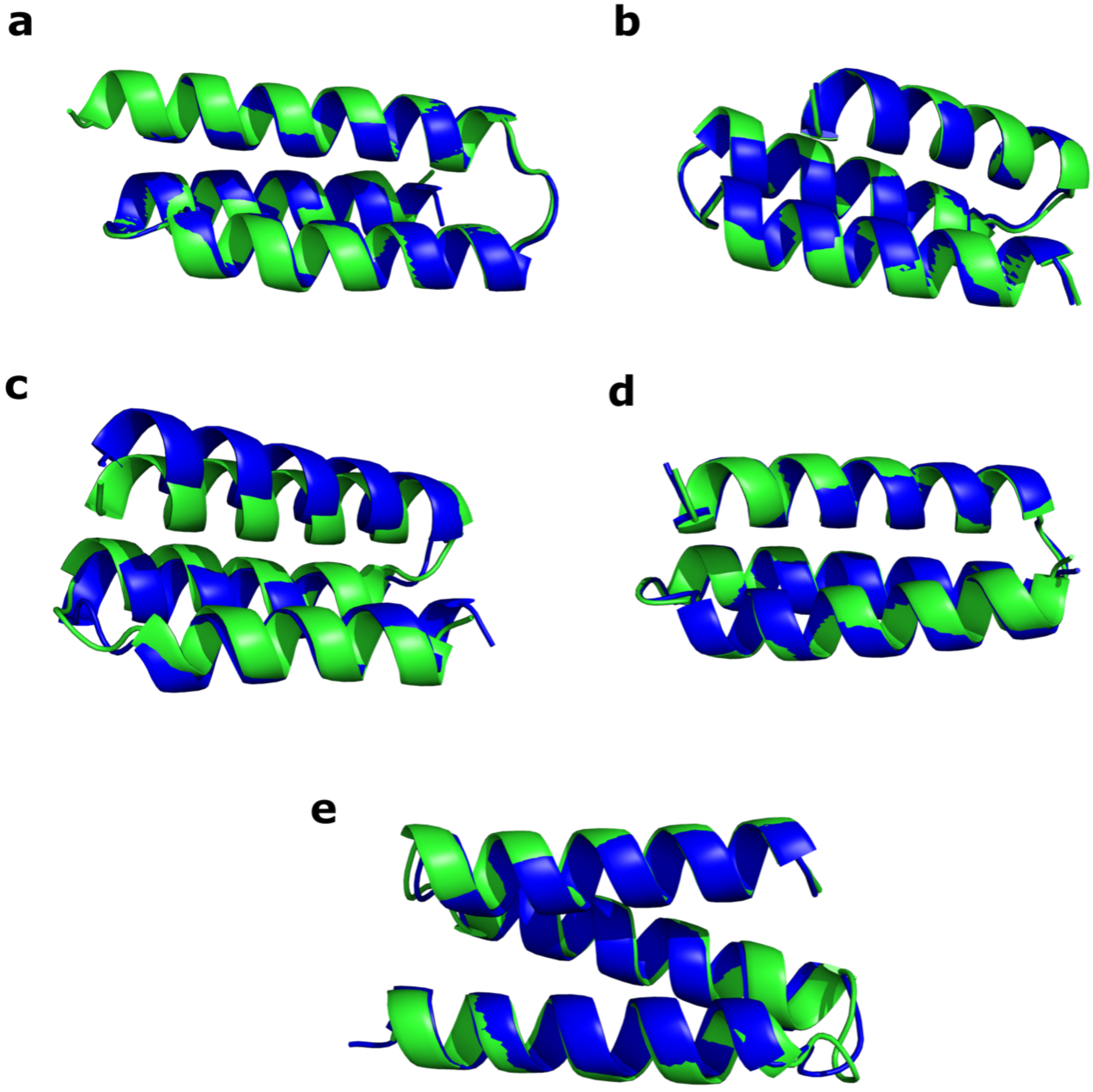
a
b
c
d
e

Supplementary Figure 2. **AF2 Monomer Predictions (Blue) are close to the Experimentally Determined Structures (Yellow). a** FGFR2 **b** IL-7Rα **c** SARS-CoV-2 Spike (LCB1) **d** SARS-CoV-2 Spike (LCB3) **e** TrkA. Source data are provided as a Source Data file.

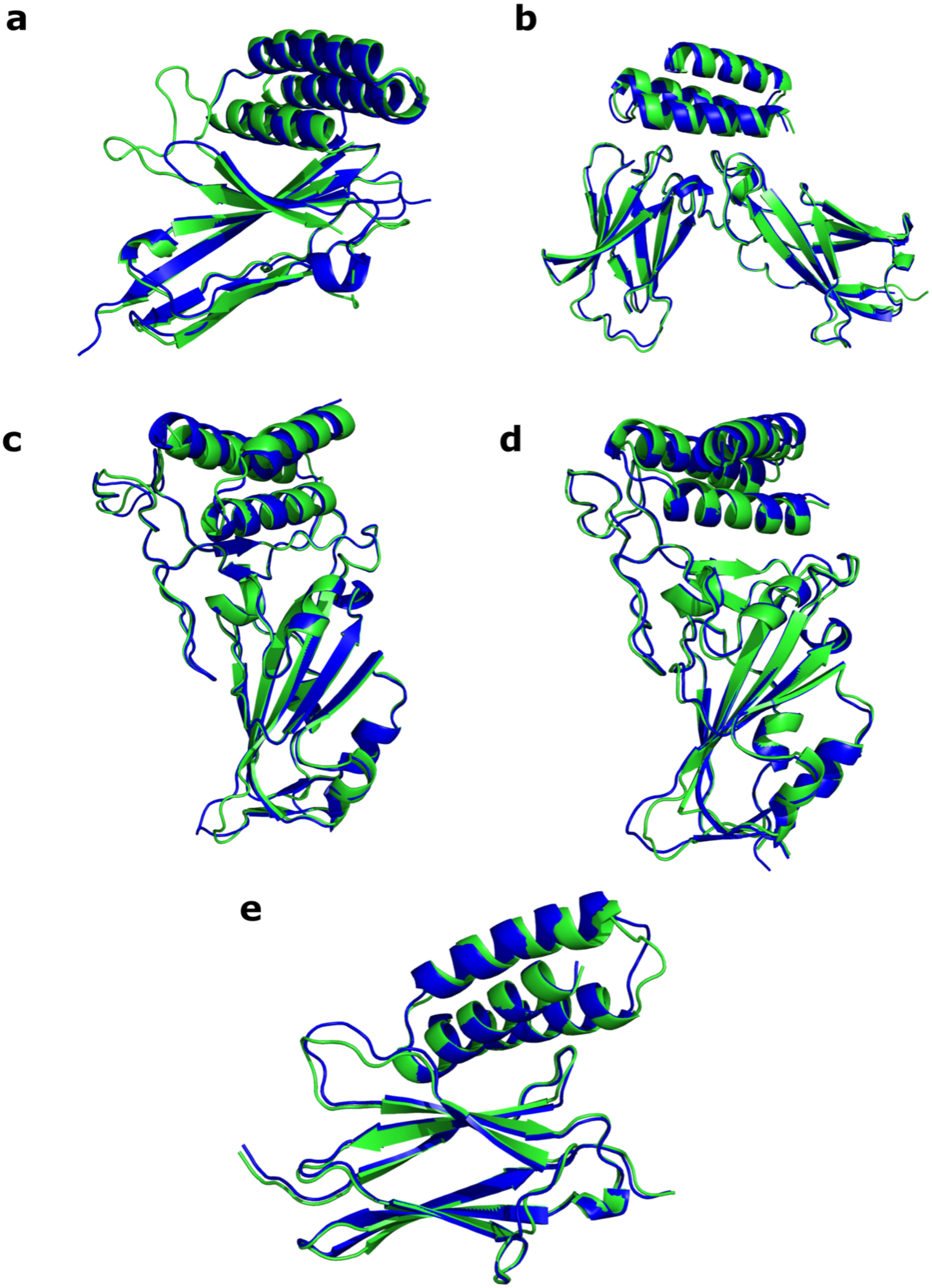
a
b
c
d
e

Supplementary Figure 3. **AF2 Complex Predictions (Blue) are close to the Experimentally Determined Structures (Yellow).** All complex predictions shown were run using AF2 with target template and the Rosetta-design model as an initial guess. **a** FGFR2 **b** IL-7Rα **c** SARS-CoV-2 Spike (LCB1) **d** SARS-CoV-2 Spike (LCB3) **e** TrkA. Source data are provided as a Source Data file.

a

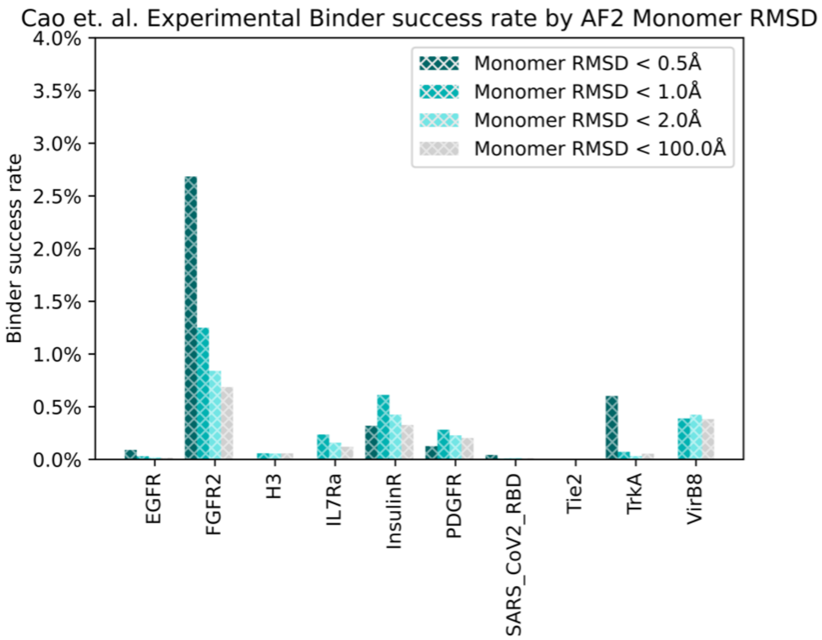
Cao et. al. Experimental Binder success rate by AF2 Monomer RMSD
Binder success rate
4.0%
3.5%
3.0%
2.5%
2.0%
1.5%
1.0%
0.5%
0.0%
Monomer RMSD < 0.5Å
Monomer RMSD < 1.0Å
Monomer RMSD < 2.0Å
Monomer RMSD < 100.0Å
EGFR
FGFR2
H3
IL7Ra
InsulinR
PDGFR
SARS_CoV2_RBD
Tie2
TrkA
VirB8

b

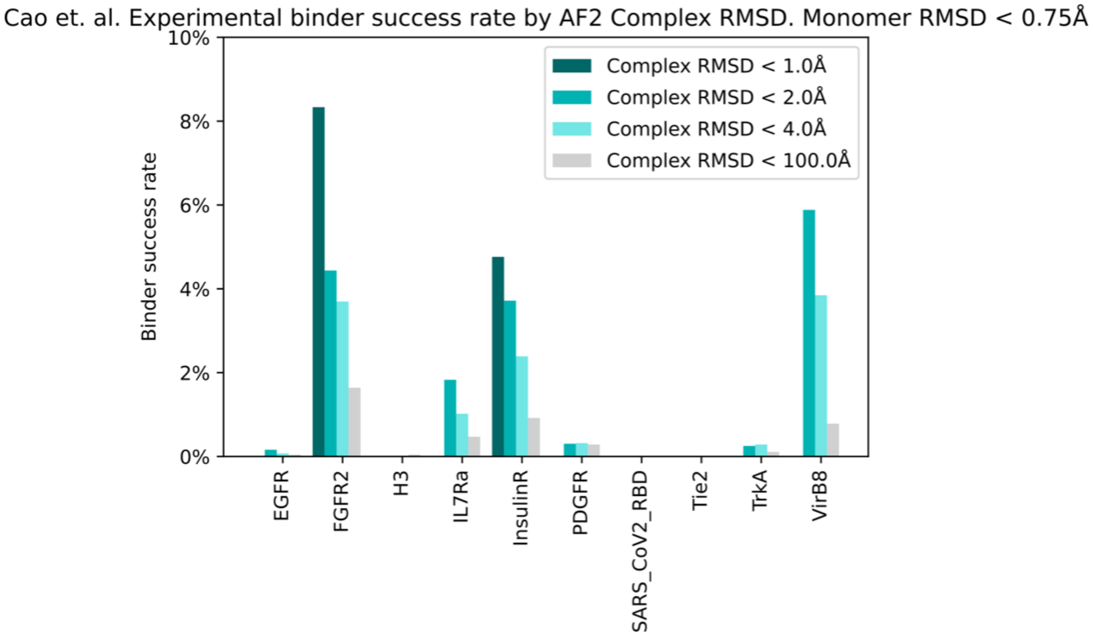
Cao et. al. Experimental binder success rate by AF2 Complex RMSD. Monomer RMSD < 0.75Å
Binder success rate
10%
8%
6%
4%
2%
0%
Complex RMSD < 1.0Å
Complex RMSD < 2.0Å
Complex RMSD < 4.0Å
Complex RMSD < 100.0Å
EGFR
FGFR2
H3
IL7Ra
InsulinR
PDGFR
SARS_CoV2_RBD
Tie2
TrkA
VirB8

Supplementary Figure 4. **Retrospective Analysis of RMSD Metrics. a** The retrospective experimental success rate (YSD SC_{50} < 4μM) for designs passing 4 thresholds of the RMSD difference between the Rosetta monomer structure and the AF2 monomer structure. **b** The retrospective experimental success rate (YSD SC_{50} < 4μM) for designs passing 4 thresholds of the RMSD difference between the Rosetta complex structure and the AF2 complex structure. Source data are provided as a Source Data file.

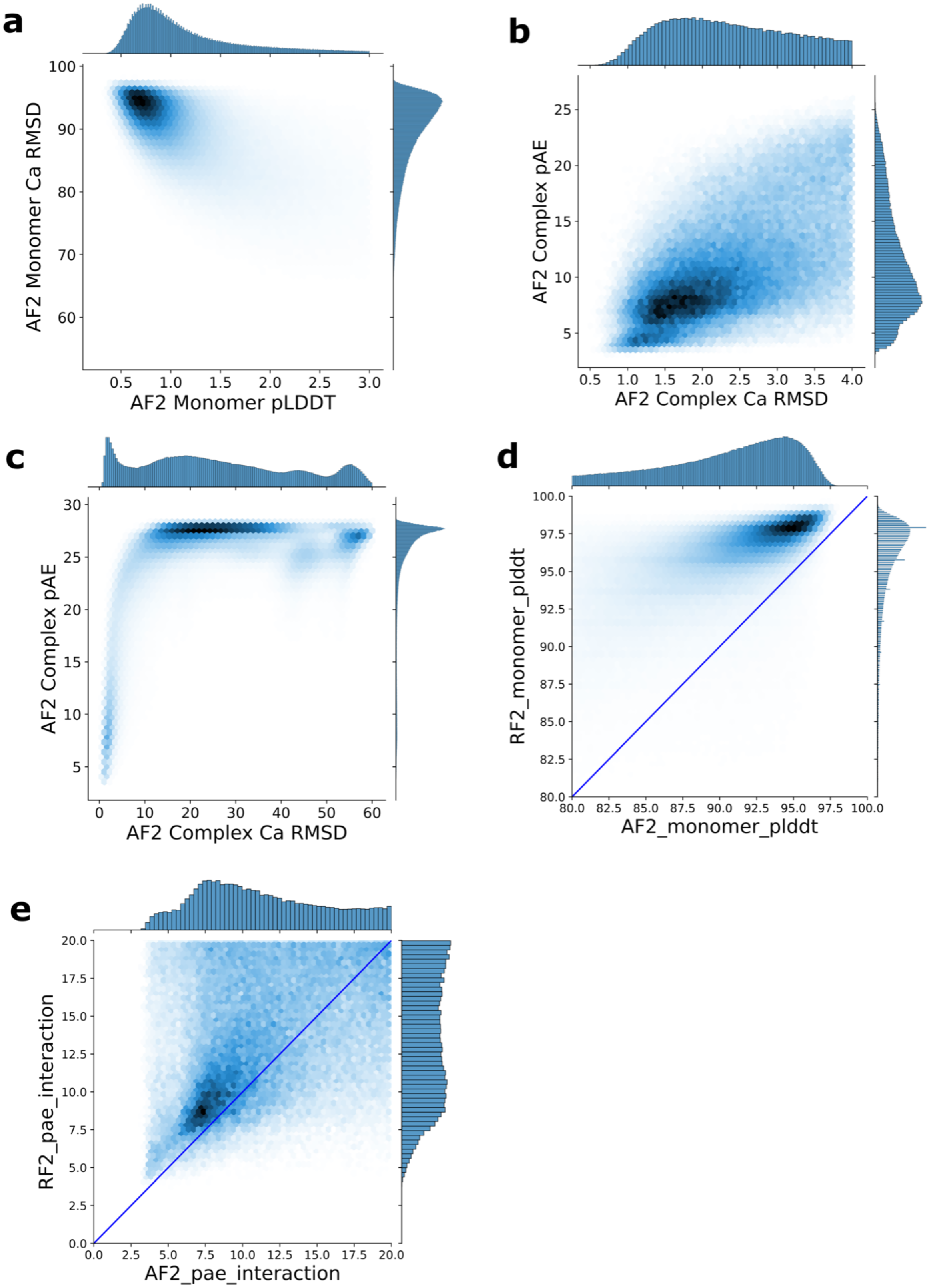
a
AF2 Monomer Ca RMSD
AF2 Monomer pLDDT
b
AF2 Complex pAE
AF2 Complex Ca RMSD
c
AF2 Complex pAE
AF2 Complex Ca RMSD
d
RF2_monomer_plddt
AF2_monomer_plddt
e
RF2_pae_interaction
AF2_pae_interaction

Supplementary Figure 5. **Correlation Between AF2 Confidence Metrics and AF2 RMSD. a** For all binders in the dataset from Cao et al. the AF2 monomer confidence (pLDDT) and the RMSD difference between the Rosetta binder structure and the AF2 binder structure is plotted. **b** For all binders in the dataset from Cao et al. the AF2 complex confidence (pAE_interaction) and the RMSD difference between the Rosetta complex structure and the AF2 complex structure is plotted. **c** Zoomed-out version of (**b**). **d** For all binders in the dataset from Cao et al. the AF2 monomer confidence (AF2_monomer_plddt) and the RF2 monomer confidence (RF2_monomer_plddt) is plotted. The line x=y is plotted in solid blue. **e** For all binders in the dataset from Cao et al. the AF2 complex confidence (AF2_pAE_interaction) and the RF2 complex confidence (RF2_pAE_interaction) is plotted. The line x=y is plotted in solid blue. Source data are provided as a Source Data file.

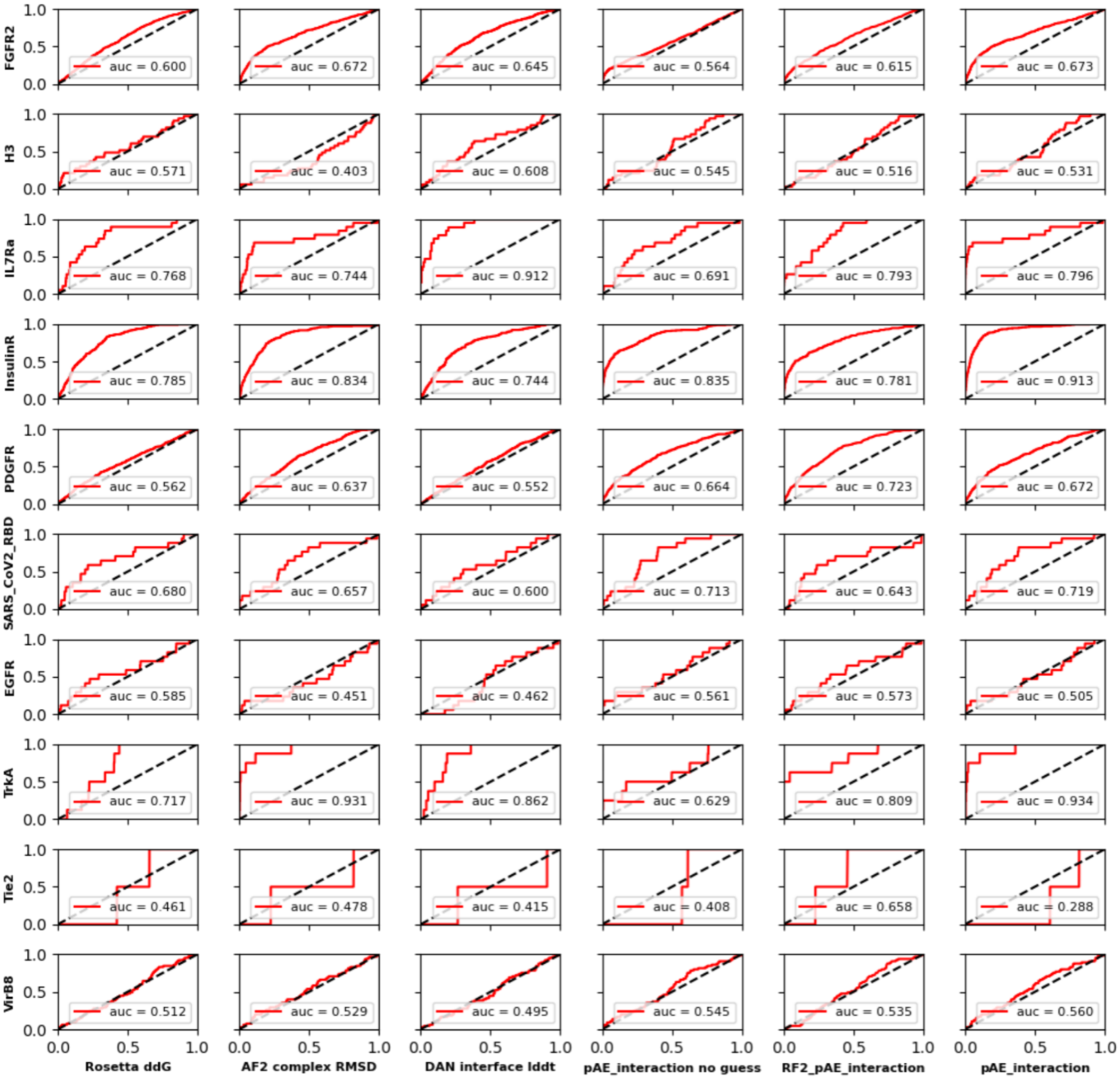
FGFR2
H3
IL7Ra
InsulinR
PDGFR
SARS_CoV2_RBD
EGFR
TrkA
Tie2
VirB8
auc = 0.600
auc = 0.672
auc = 0.645
auc = 0.564
auc = 0.615
auc = 0.673
auc = 0.571
auc = 0.403
auc = 0.608
auc = 0.545
auc = 0.516
auc = 0.531
auc = 0.768
auc = 0.744
auc = 0.912
auc = 0.691
auc = 0.793
auc = 0.796
auc = 0.785
auc = 0.834
auc = 0.744
auc = 0.835
auc = 0.781
auc = 0.913
auc = 0.562
auc = 0.637
auc = 0.552
auc = 0.664
auc = 0.723
auc = 0.672
auc = 0.680
auc = 0.657
auc = 0.600
auc = 0.713
auc = 0.643
auc = 0.719
auc = 0.585
auc = 0.451
auc = 0.462
auc = 0.561
auc = 0.573
auc = 0.505
auc = 0.717
auc = 0.931
auc = 0.862
auc = 0.629
auc = 0.809
auc = 0.934
auc = 0.461
auc = 0.478
auc = 0.415
auc = 0.408
auc = 0.658
auc = 0.288
auc = 0.512
auc = 0.529
auc = 0.495
auc = 0.545
auc = 0.535
auc = 0.560
Rosetta ddG
AF2 complex RMSD
DAN interface lddt
pAE_interaction no guess
RF2_pAE_interaction
pAE_interaction

Supplementary Figure 6. **Retrospective Analysis of Complex Metrics.** For each combination of 10 targets and 6 interface metrics a Receiver-Operator Characteristic curve quantifying the discriminatory power of the metric to separate successful designs (SC_{50} < 4 μM) from unsuccessful designs (SC_{50} >= 4 μM) is plotted. The True Positive Rate is plotted on the y-axis and the False Positive Rate on the x-axis. The Area Under the Curve (AUC) is included for each plot. Source data are provided as a Source Data file.

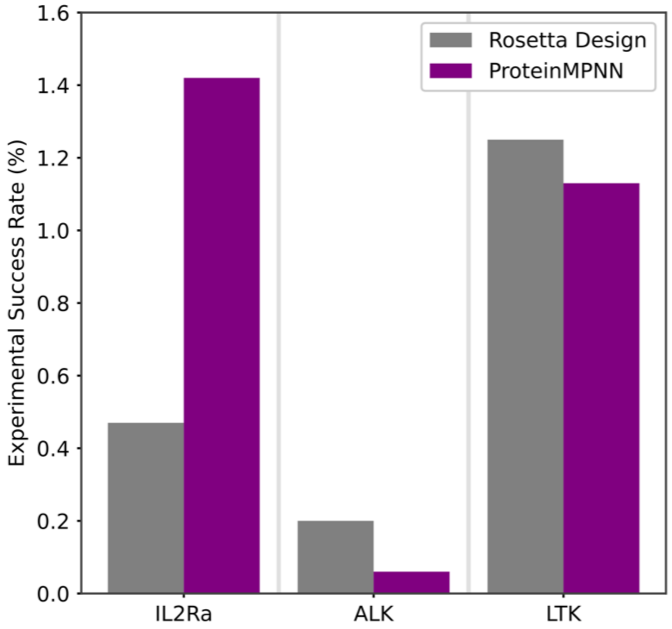

Supplementary Figure 7. **Experimental Success Rate of Binders Generated by ProteinMPNN versus Rosetta Design.** The experimental success rate for libraries generated by redesign of DL filtered designs with Rosetta FastDesign versus ProteinMPNN and filtered by DL-based filtering for three prospective targets. Source data are provided as a Source Data file.

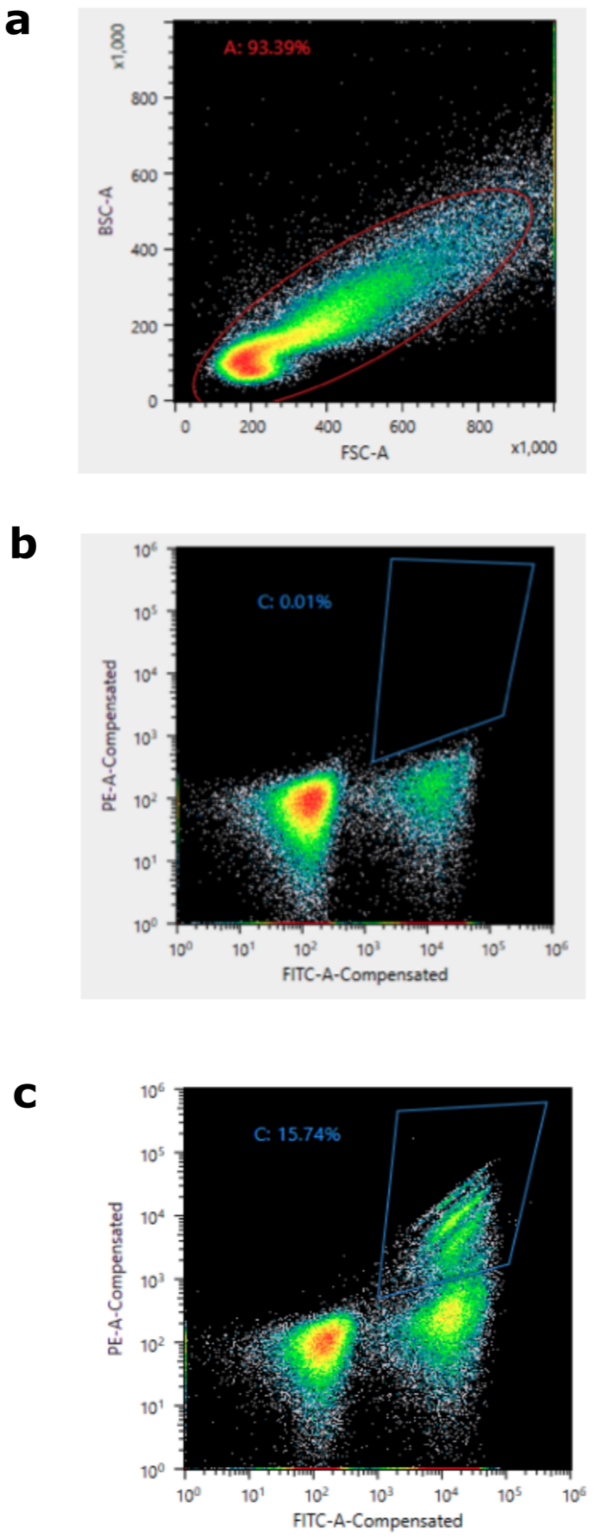
a
A: 93.39%
BSC-A
x1,000
FSC-A
x1,000
b
C: 0.01%
PE-A-Compensated
FITC-A-Compensated
c
C: 15.74%
PE-A-Compensated
FITC-A-Compensated

Supplementary Figure 8. **Gating Strategy for FACS Sorting of ALK Binder Library. a** Forward scatter (FSC) versus back scatter (BSC) plot with gate used in red. **b** Negative sort with gate shown in blue. **c** Binding sort with gate (same gate as used in (**b**)) shown in blue. Source data are provided as a Source Data file.

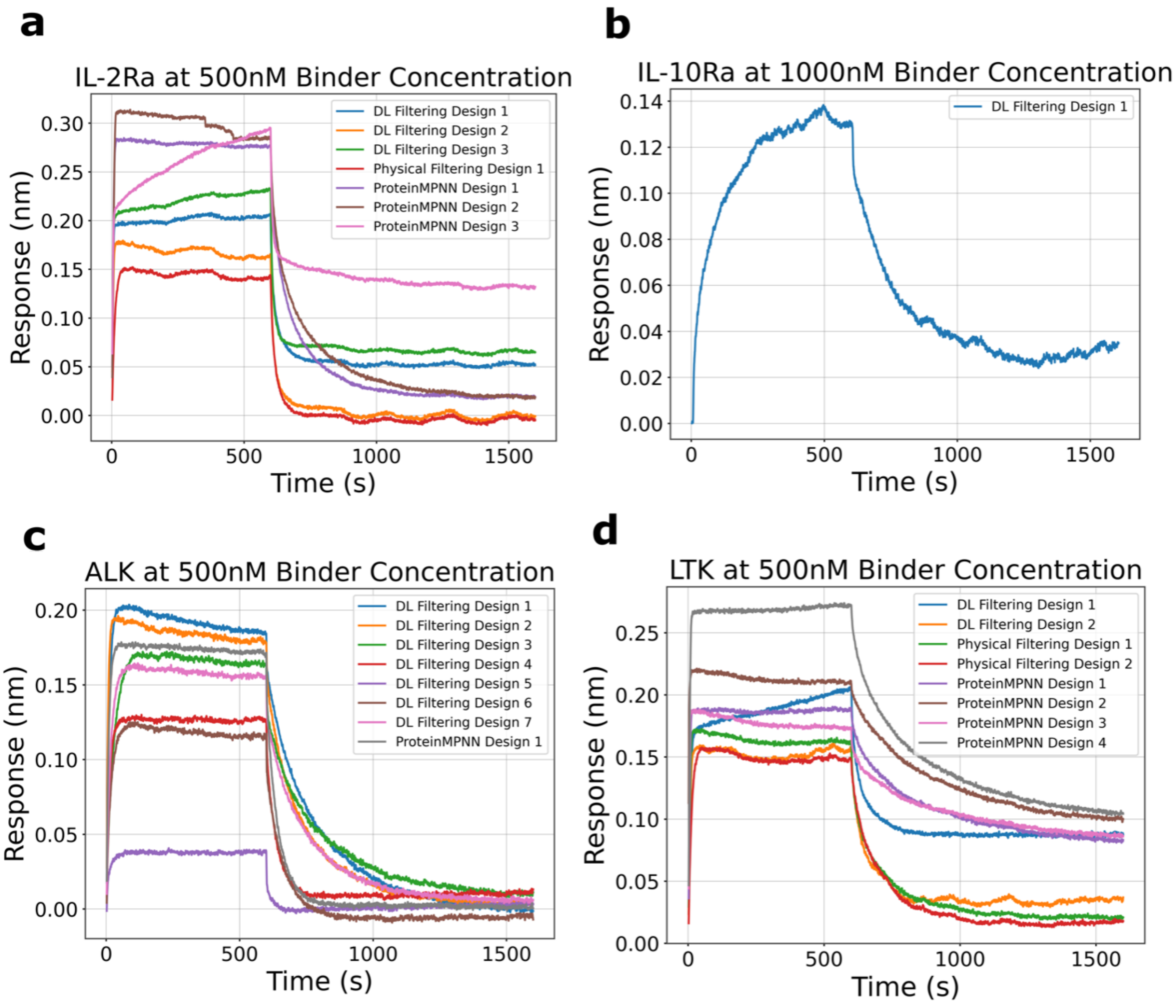

Supplementary Figure 9. **Biophysical Characterization of Select Designs.** For each target, 7-8 designs to that target, which showed binding by Yeast Surface Display, were screened for binding by single-concentration Biolayer Interferometry (BLI). For IL-10Rɑ only one design was identified and that is the only design which was screened by BLI. All designs tested showed binding signal by BLI. No successful ProteinMPNN designs against IL-10Rɑ were identified and only one successful ProteinMPNN design was identified to bind ALK. Source data are provided as a Source Data file.
