# Supplementary material for: Improving de novo protein binder design with deep learning
Source: Nat Commun. 2023 May 6;14:2625. doi: 10.1038/s41467-023-38328-5 (PMC10163288; doi:10.1038/s41467-023-38328-5)
Supplement: Supplementary file 3 — Reporting Summary [file 41467_2023_38328_MOESM3_ESM.pdf]

nature research

Corresponding author(s): David Baker

Last updated by author(s): Mar 16, 2023

Reporting Summary

Nature Research wishes to improve the reproducibility of the work that we publish. This form provides structure for consistency and transparency in reporting. For further information on Nature Research policies, see our Editorial Policies and the Editorial Policy Checklist.

Statistics

For all statistical analyses, confirm that the following items are present in the figure legend, table legend, main text, or Methods section.

n/a	Confirmed	
☐	☒	The exact sample size (*n*) for each experimental group/condition, given as a discrete number and unit of measurement
☒	☐	A statement on whether measurements were taken from distinct samples or whether the same sample was measured repeatedly
☒	☐	The statistical test(s) used AND whether they are one- or two-sided<br>*Only common tests should be described solely by name; describe more complex techniques in the Methods section.*
☒	☐	A description of all covariates tested
☒	☐	A description of any assumptions or corrections, such as tests of normality and adjustment for multiple comparisons
☒	☐	A full description of the statistical parameters including central tendency (e.g. means) or other basic estimates (e.g. regression coefficient) AND variation (e.g. standard deviation) or associated estimates of uncertainty (e.g. confidence intervals)
☒	☐	For null hypothesis testing, the test statistic (e.g. *F*, *t*, *r*) with confidence intervals, effect sizes, degrees of freedom and *P* value noted<br>*Give P values as exact values whenever suitable.*
☒	☐	For Bayesian analysis, information on the choice of priors and Markov chain Monte Carlo settings
☒	☐	For hierarchical and complex designs, identification of the appropriate level for tests and full reporting of outcomes
☒	☐	Estimates of effect sizes (e.g. Cohen's *d*, Pearson's *r*), indicating how they were calculated

Our web collection on statistics for biologists contains articles on many of the points above.

Software and code

Policy information about availability of computer code

Data collection	We use the Sony SH800S with software version 2.1.5 for cell sorting. We use the Illumina NextSeq for high throughput DNA sequencing. We use the Octet Red96 and Octet R8 with Octet BLI Discovery 12.2.1.18 software for biolayer interferometry measurements.
Data analysis	Python 3.7, DNAWorks 2.0, ProteinMPNN v1.0.1, AlphaFold v2.0.1, https://github.com/nrbennet/dl_binder_design v1.0.0

For manuscripts utilizing custom algorithms or software that are central to the research but not yet described in published literature, software must be made available to editors and reviewers. We strongly encourage code deposition in a community repository (e.g. GitHub). See the Nature Research guidelines for submitting code & software for further information.

Data

Policy information about availability of data

All manuscripts must include a data availability statement. This statement should provide the following information, where applicable:

- Accession codes, unique identifiers, or web links for publicly available datasets
- A list of figures that have associated raw data
- A description of any restrictions on data availability

Source data are provided with this paper. The raw data from the prospective study, the raw scores of the retrospective analysis, the input structures and benchmarking scores for the efficiency study, and the raw data from the biolayer interferometry measurements are available at the following repository hosted by the Institute for Protein Design:

The main supplement (136 MB)
  Contains these files:
    design_models_final_combo_optimized/

design_models_sequence/
design_models_ssm_natives/
design_stats/
dna_production_scripts/
figure_data/
ngs_analysis_scripts/

files.ipd.uw.edu/pub/improving_dl_binders_2023/supplemental_files/scripts_and_main_pdbs.tar.gz

Experimental data and data derived from that data (155 MB)
Contains these files:
ngs_data/
ngs_data_analysis/

files.ipd.uw.edu/pub/improving_dl_binders_2023/supplemental_files/experimental_data_and_analysis.tar.gz

All ordered proteins in .pdb.gz format: (~100K files; 15 GB)
Contains these files:
design_models_pdbs/

files.ipd.uw.edu/pub/improving_dl_binders_2023/supplemental_files/design_models_pdb.tar.gz

All ordered proteins in Rosetta binary silent format (6.1 GB)
Contains these files:
design_models_silent/

files.ipd.uw.edu/pub/improving_dl_binders_2023/supplemental_files/design_models_silent.tar.gz

The docks we used for the efficiency benchmark (6.1 GB)
Contains these files:
efficiency_benchmark_docks/

files.ipd.uw.edu/pub/improving_dl_binders_2023/supplemental_files/efficiency_benchmark_docks.tar.gz

Field-specific reporting

Please select the one below that is the best fit for your research. If you are not sure, read the appropriate sections before making your selection.

☒ Life sciences ☐ Behavioural & social sciences ☐ Ecological, evolutionary & environmental sciences

For a reference copy of the document with all sections, see nature.com/documents/nr-reporting-summary-flat.pdf

Life sciences study design

All studies must disclose on these points even when the disclosure is negative.

Sample size	For the success rate comparison shown in Figure 2, we ordered 30,000 designs for each target. We included all designs which passed the AF2 cutoffs in silico and then selected Rosetta-filtering designs to bring the total number per target to 30,000. No statistical methods were used to determine sample size.
Data exclusions	No data were excluded from the analyses.
Replication	No attempt at replication was made.
Randomization	The experiments were not randomized.
Blinding	The Investigators were not blinded to allocation during experiments and outcome assessment.

Reporting for specific materials, systems and methods

We require information from authors about some types of materials, experimental systems and methods used in many studies. Here, indicate whether each material, system or method listed is relevant to your study. If you are not sure if a list item applies to your research, read the appropriate section before selecting a response.

Materials & experimental systems

n/a	Involved in the study
☒	☐ Antibodies
☒	☐ Eukaryotic cell lines
☒	☐ Palaeontology and archaeology
☒	☐ Animals and other organisms
☒	☐ Human research participants
☒	☐ Clinical data
☒	☐ Dual use research of concern

Methods

n/a	Involved in the study
☒	☐ ChIP-seq
☐	☒ Flow cytometry
☒	☐ MRI-based neuroimaging

Flow Cytometry

Plots

Confirm that:

☒ The axis labels state the marker and fluorochrome used (e.g. CD4-FITC).

☒ The axis scales are clearly visible. Include numbers along axes only for bottom left plot of group (a 'group' is an analysis of identical markers).

☒ All plots are contour plots with outliers or pseudocolor plots.

☒ A numerical value for number of cells or percentage (with statistics) is provided.

Methodology

Sample preparation	Sample preparation was done as described in Cao et al. (2022)
Instrument	Sony SH800S
Software	Sorting was done as described in Cao et al. (2022)
Cell population abundance	The percentage of sequences that match the designed binder in each NGS sample was analyzed. The percentage ranges between 50-90%.
Gating strategy	The FSC/BSC gate is selected to select single living yeast cells. The positive gate for flow cytometry is selected to select cells which express both the binder protein and show binding signal above the negative sort (see Supplementary Figure 8).

☒ Tick this box to confirm that a figure exemplifying the gating strategy is provided in the Supplementary Information.
