# Supplementary figures and images for: Improving de novo protein binder design with deep learning
Source: Nat Commun. 2023 May 6;14:2625. doi: 10.1038/s41467-023-38328-5 (PMC10163288; doi:10.1038/s41467-023-38328-5)

## Slide 1
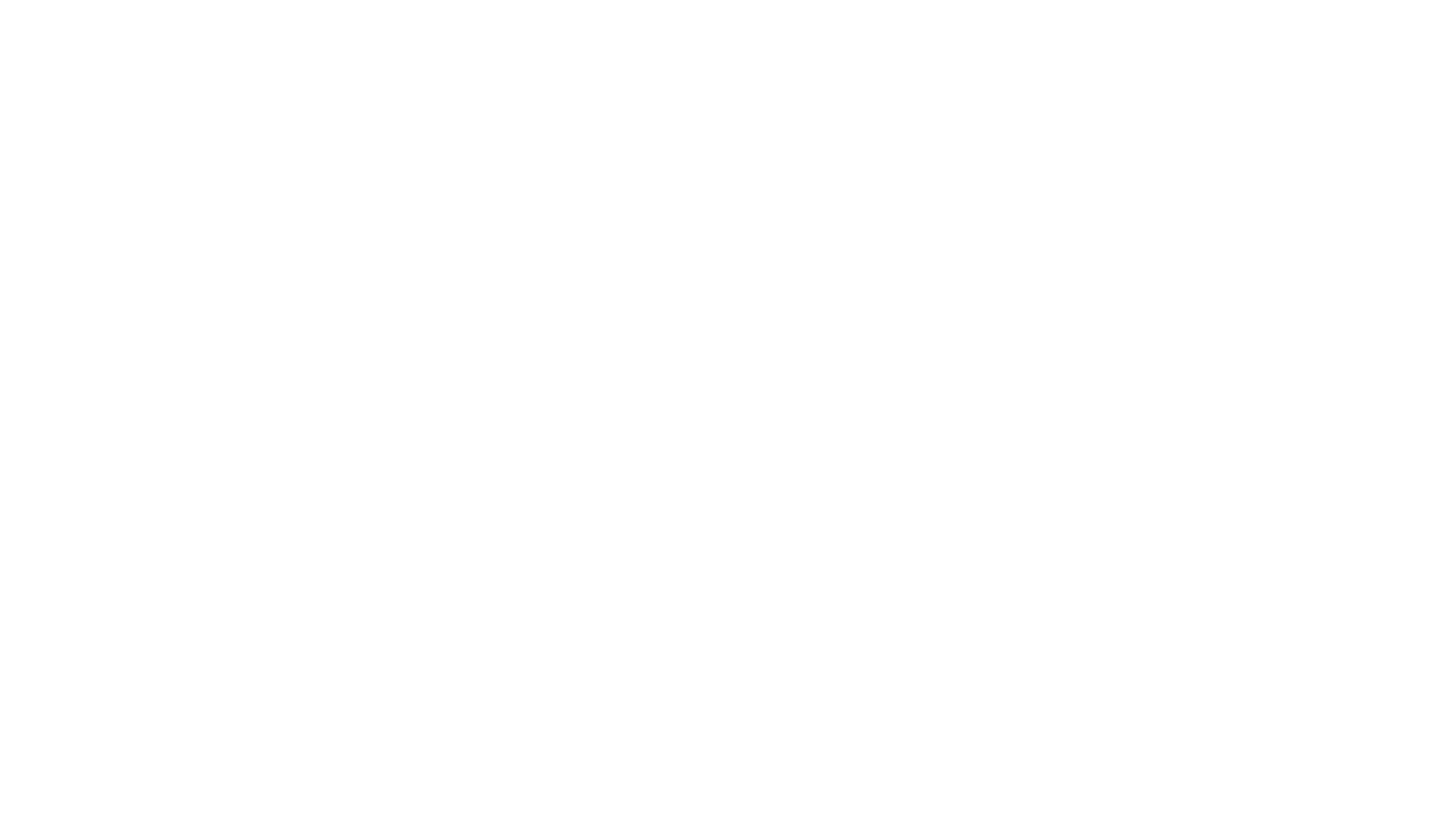

#

## Slide 2
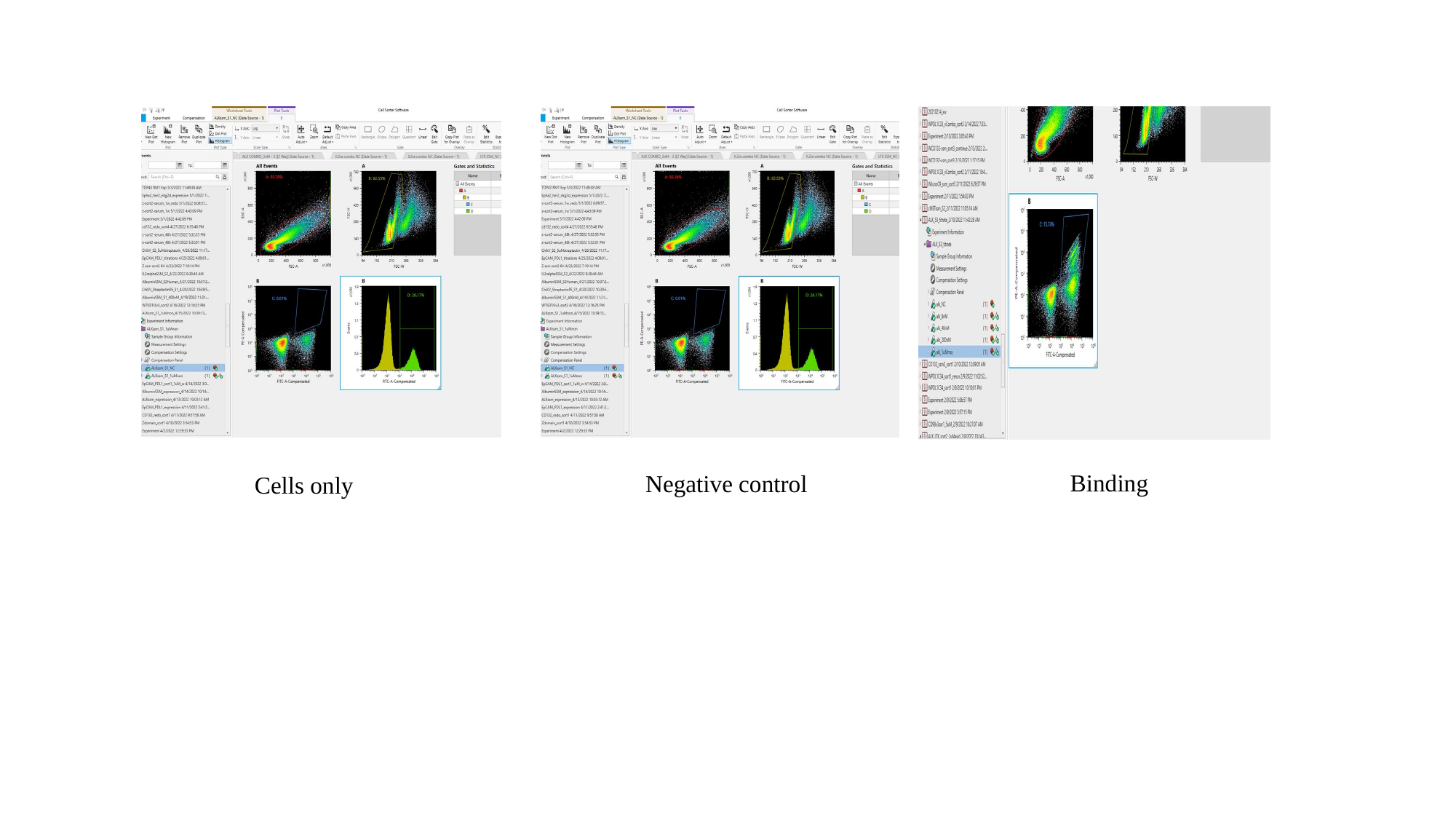

Binding
Negative control
Cells only

Supplement: Supplementary file 4 — Source Data File [file 41467_2023_38328_MOESM4_ESM.zip › all_data/YSD_gates/example_FACS_gates.pptx]
